# Visualization and quantification of the degenerative pattern of the talus in unilateral varus ankle osteoarthritis

**DOI:** 10.1038/s41598-019-53746-6

**Published:** 2019-11-25

**Authors:** Hiroyuki Seki, Naomichi Ogihara, Tetsuro Kokubo, Yasunori Suda, Ken Ishii, Takeo Nagura

**Affiliations:** 1 Department of Clinical Biomechanics, Keio University, 35 Shinano-machi, Shinjuku-ku, Tokyo 160-8582 Japan; 2 Department of Orthopeadic Surgery, International University of Health and Welfare Mita Hospital, 1-4-3 Mita, Minato-ku, Tokyo 108-8329 Japan; 3 Department of Orthopeadic Surgery, School of Medicine, International University of Health and Welfare, 4-3 Kouzunomori, Narita-shi, Chiba 107-0062 Japan; 4 Department of Mechanical Engineering, Faculty of Science and Technology, Keio University, 3-14-1 Hiyoshi, Kohoku-ku, Yokohama-shi, Kanagawa 223-8522 Japan; 5 Department of Biological Sciences, Graduate School of Science, The University of Tokyo, 7-3-1, Hongo, Bunkyo-ku, Tokyo 113-0033 Japan; 6 Department of Orthopeadic Surgery, Tachikawa Hospital, 4-2-22 Nishiki-cho, Tachikawa-shi, Tokyo 190-8531 Japan; 7 Department of Orthopeadic Surgery, International University of Health and Welfare Shioya Hospital, 77 Tomita, Yaita-shi, Tochigi 329-2145 Japan

**Keywords:** Targeted bone remodelling, Bone

## Abstract

The aim of this study was to quantify and visualize the degenerative patterns of the talus in ankle osteoarthritis (OA). The differences in talar morphology between sides of patients with unilateral varus ankle OA (medial talar tilt > 4°) were compared. Computed tomography images of both feet of 35 patients (OA: 22 patients, control: 13 patients) were analyzed. Each surface model of the right and left tali was registered to the opposite talus via a mirror-image technique and an iterative closest point algorithm. The surface deviation between the two models was quantified and visualized by deviation color maps. The results quantitatively demonstrated that osteophytes are generated in the area under the antero-medial margin of the trochlea in OA tali. In severe OA tali, bone resorption of more than 2 mm in the medial portion of the trochlea, as well as a similar degree of osteophyte formation on the lateral surface, was also seen. Stereotypical patterns of degeneration occurring in OA tali were successfully visualized and quantified by left-right comparison of patients with unilateral ankle OA. Such information would contribute to better understanding of the development of ankle OA and preoperative planning of total ankle arthroplasty and arthrodesis.

## Introduction

Ankle osteoarthritis (OA) is one of the most important progressive diseases causing pain and chronic disability in the ankle joint^1^. In the majority of such ankle OA patients, not only bony degeneration of the talus, but also tibiotalar varus alignment is observed, regardless of underlying etiology^2^. Such bony degeneration and joint alignment have been clinically assessed based on a plain weight-bearing radiograph^3,4^. However, morphological assessment of ankle OA based on a two-dimensional radiograph is generally difficult due to the fact that the talus is overlapped with the tibia, fibula, navicular, and calcaneus; thus, the talar bony degeneration is not clearly visible on a plain radiograph.

Recently, computed tomography (CT) examinations have been used to observe the three-dimensional bony degeneration of talar bodies in patients with ankle OA^5,6^. In these previous studies, the shape of the OA talus was compared with that of subjects without OA. However, owing to the marked inter-individual variations in the size and shape of the talus, detailed analysis of the morphological degeneration of the talus due to ankle OA has been challenging. Therefore, the patterns of morphological degeneration of the talus due to ankle OA have not been quantified previously.

If we analyze the left-right differences in talar morphology of patients with unilateral varus ankle OA, however, the effect of the large inter-individual variabilities of the talar morphology can be eliminated, and the pattern of morphological degeneration of the talar morphology in patients with ankle OA can be extracted. Therefore, the present study investigated the left-right differences in talar morphology of patients with unilateral varus ankle OA based on CT imaging, with the aim of extracting the pattern of bony degeneration of the talus in patients with ankle OA. Specifically, the left and mirror-reflected right talar surface models were generated and registered to each other to quantify the bony differences of the OA talus with respect to the opposite talus without OA (non-OA). It has been demonstrated that left and right tali of healthy human ankle joints show a strong degree of symmetry^7^. Therefore, an excessive left-right difference in the morphology of the talus observed in such patients could possibly be attributed to osteophyte formation and bone resorption due to ankle OA. Quantitative understanding of the three-dimensional degeneration of the talus in patients with ankle OA is important for foot and ankle orthopedic surgeons for correct preoperative planning and achieving successful clinical outcomes of total ankle arthroplasty^8^ and arthrodesis^9^.

## Results

Based on the plain radiographs, 22 patients of the OA group were found to be in stage 3a, 3b, or 4 on the OA side and in stage 1 or less on the opposite non-OA side, according to the Takakura classification^10^. The numbers of patients classified into each stage were five, eleven, and six, respectively. Thirteen patients of the control group were not diagnosed as having ankle OA on plain radiographs. There were no significant differences between the two groups in age and the sex ratio.

**Figure 1:**
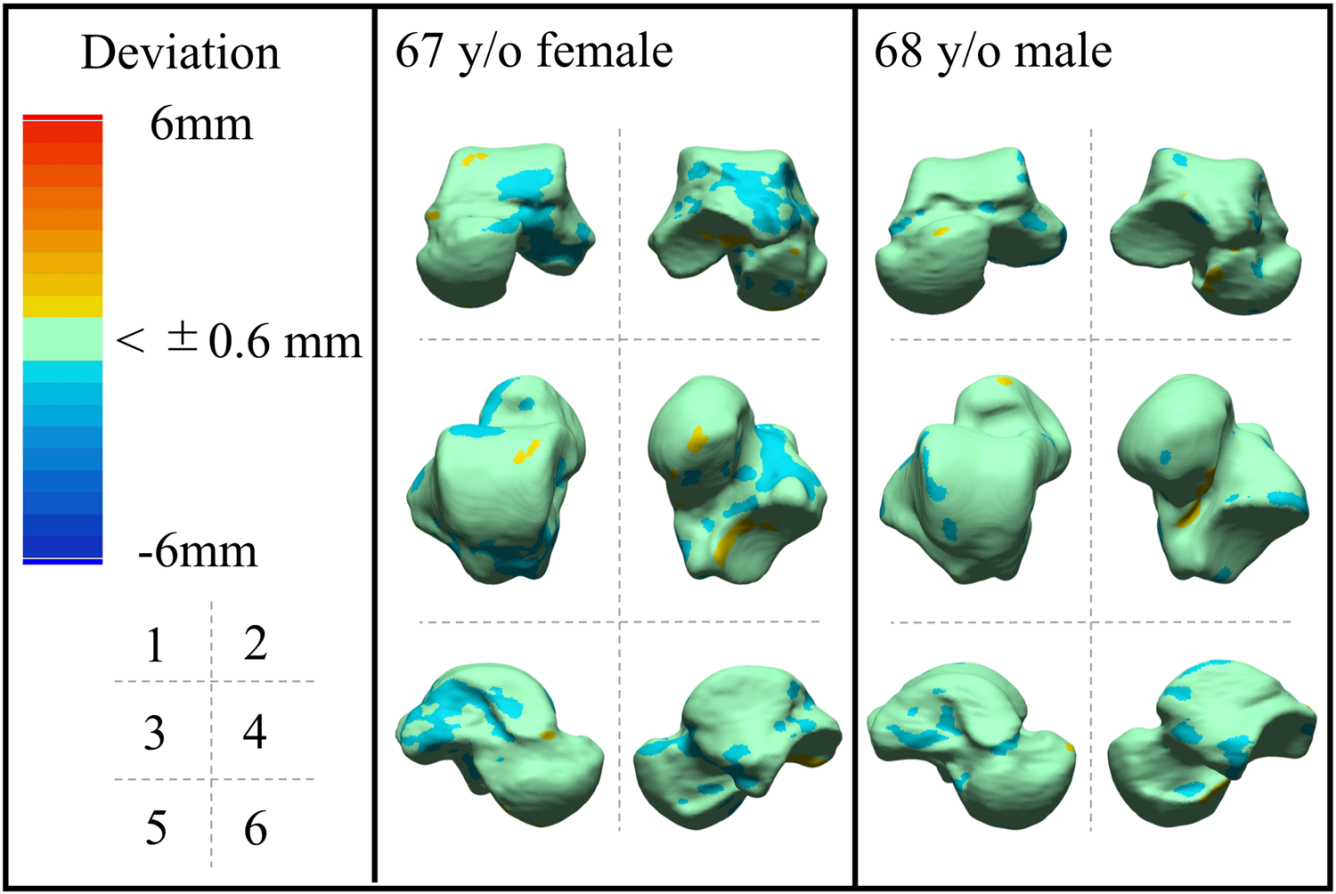
Deviation color maps of left-right comparisons of the tali in the control group. Two representative examples are presented. 1. Anterior view. 2. Posterior view. 3. Superior view. 4. Inferior view. 5. Medial view. 6. Lateral view. y/o = years old.

**Figure 2:**
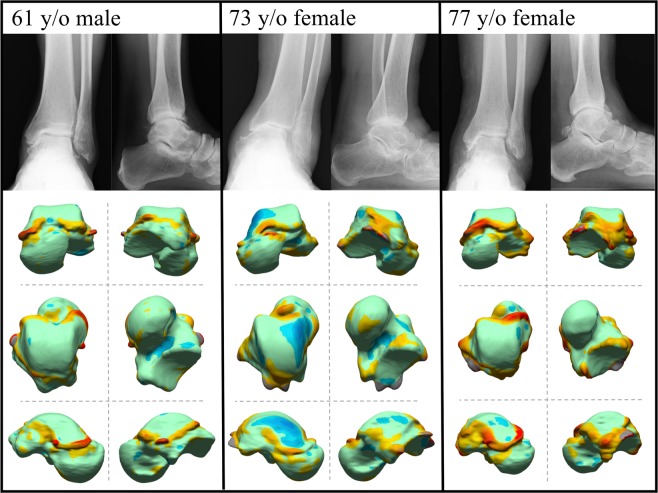
Plain radiographs and deviation color maps of the osteoarthritis (OA) groups in stage 3a. Deviation color maps of three representative examples are presented. The red and blue colors are deviations of the OA talus outside and inside of the healthy talus. See Fig. 1 and text for more details.

**Figure 3:**
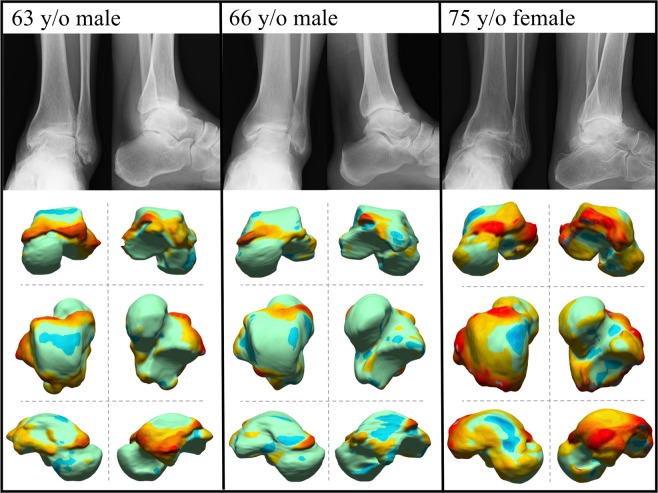
Plain radiographs and deviation color maps of the OA groups in stage 3b. Deviation color maps of three representative examples are presented. The red and blue colors are deviations of the OA talus outside and inside of the healthy talus. See Fig. 1 and text for more details.

**Figure 4:**
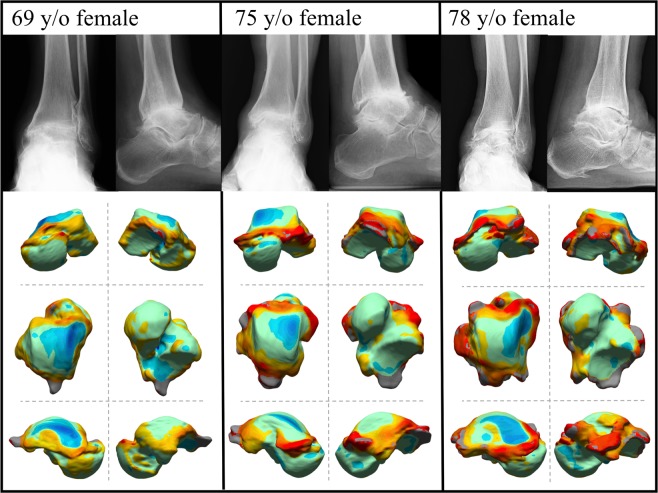
Plain radiographs and deviation color maps of the OA groups in stage 4. Deviation color maps of three representative examples are presented. The red and blue colors are deviations of the OA talus outside and inside of the healthy talus. See Fig. 1 and text for more details.

The deviation color maps of the control group indicated that the tali were essentially bilaterally symmetrical in the control group (Fig. 1). On the other hand, the deviation color map of stage 3a showed some osteophyte formation along the anteromedial margin of the trochlea articular surface. (Fig. 2) In stage 3b OA tali, osteophyte formation around the lateral process was also observed. (Fig. 3) In stage 4 OA tali, bone resorption on the anteromedial trochlear articular surface was observed (Fig. 4), indicating that the trochlear articular surface of OA tali inclined medially on the coronal plane. However, deformation of posterior calcaneal articular surfaces was found to be relatively minor, regardless of the severity of ankle OA (Figs. 2–4).

**Figure 5:**
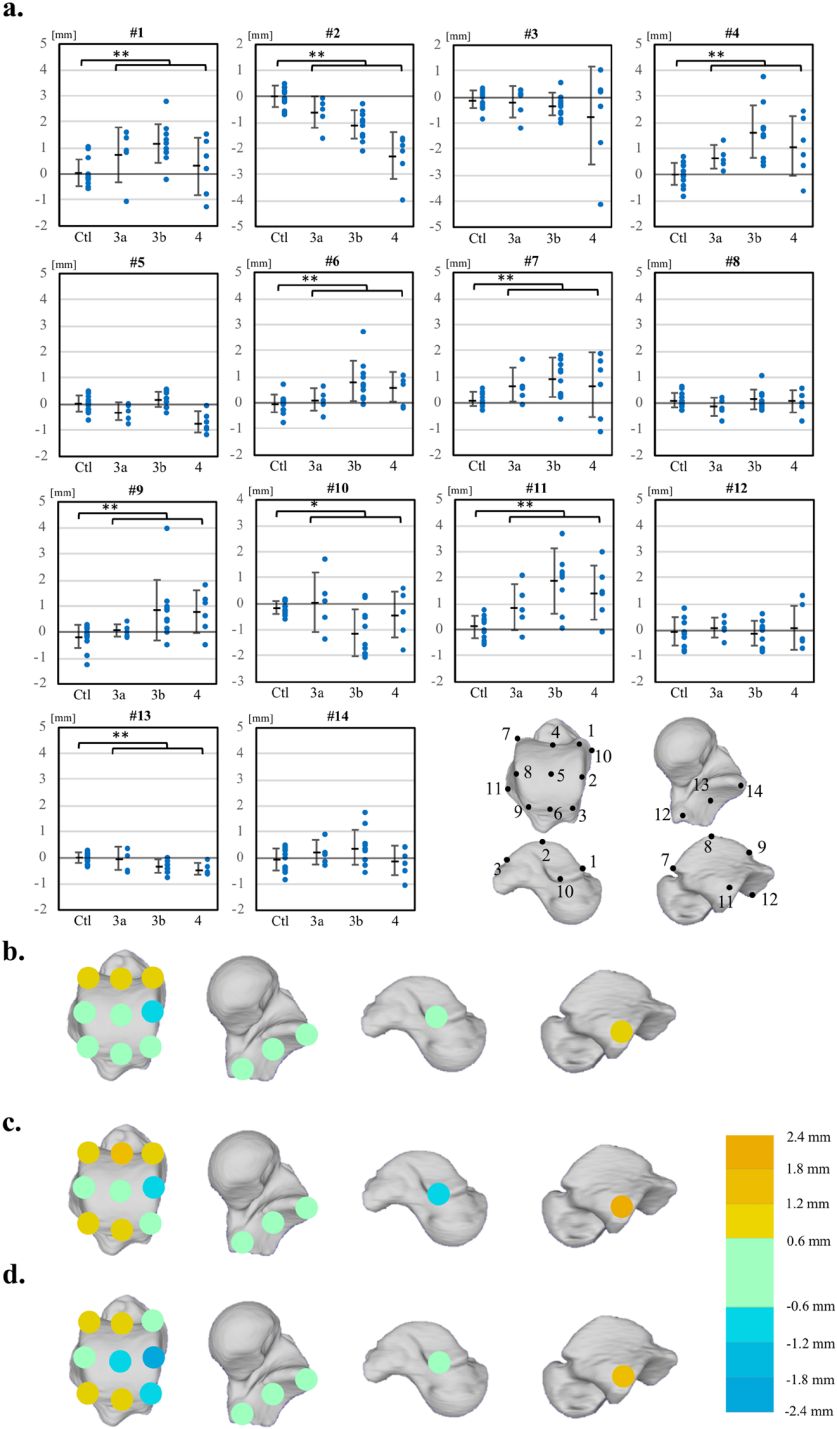
The amounts of surface deviations in the OA and control groups at 14 regions defined on the talar articular surfaces (**a**), and the color map of the mean deviations for stages 3a (**b**), 3b (**c**), and 4(**d**). The 14 landmarks used to define the 14 regions are: #1, the most anterior point of the medial margin of the trochlea; #2, the most superior point of the medial margin of the trochlea; #3, the most posterior point of the medial margin of the trochlea; #4, the midpoint of the anterior margin of the trochlea; #5, the most superior point of the midsagittal curve of the trochlea; #6, the midpoint of the posterior margin of the trochlea; #7, the most anterior point on the lateral margin of the trochlea; #8, the most superior point of the lateral margin of the trochlea; #9, the most posterior point on the lateral margin of the trochlea; #10, the most inferior point of the medial articular facet; #11, the most inferior point of the lateral articular facet; #12, the most proximo-medial point of the posterior calcaneal articular surface; #13, the centroid of the posterior calcaneal articular surface; #14, the disto-lateral point of the posterior calcaneal articular surface. Error bars indicate standard deviations. The deviations are positive if the surface of the OA talus is outside the opposite non-OA talus, and negative if the surface is inside. **p < 0.05 and power > 0.8. *p < 0.05 and power > 0.4.

Figure 5 shows the amounts of surface deviation in the OA and control groups at 14 regions defined on the talar articular surfaces (See Methods). It was observed that the amount of osteophyte formation was largest at the lateral facet of the trochlea (#11), with deviation of approximately 2 mm in severe varus ankle OA, followed by the anterior margins of the trochlea (#1, 4, 7), with deviation of 1 to 2 mm. The amount of bone resorption was largest at the medial margin of the trochlea (#2), with mean deviation of more than 2 mm in severe varus ankle OA.

## Discussion

The present study successfully extracted and visualized the morphological differences of talar articular morphology in patients with unilateral varus ankle OA. The previous three-dimensional morphological analyses of ankle OA compared differences in talar morphology between OA patients and healthy subjects^5,6^. However, the human talus generally shows marked inter-individual variations in size and shape^7^. Therefore, talar morphological differences extracted in these studies were the result of not only degeneration due to ankle OA, but also to possible differences in the pattern of congenital inter-individual variations. On the other hand, the present study compared the right and left tali of the same individual in patients with unilateral varus ankle OA to extract pure morphological differences of the talus due to ankle OA, while eliminating the effects of inter-individual variations. To the best of our knowledge, this is the first study to visualize and quantify the pattern of osteophyte formation and bone resorption of tali in ankle OA. This information would be very useful for the operative technique of bone recession for the talus component in total ankle arthroplasty and arthrodesis.

In the present study, there were significant left-right differences in talar morphology in patients with unilateral ankle OA due to osteophyte formation and bone resorption, but this was not the case in the control group, as demonstrated previously^7^. In particular, the talar spur was found to protrude medially off the medial edge of the talar neck even in the early stage of ankle OA (Figs. 2–4). This finding is consistent with the results of arthroscopy-based^11,12^ and CT-based^13^ morphological analyses of the talus in ankle anterior impingement syndrome or chronic ankle instability patients. This correspondence may indicate that these ankle diseases could be regarded as a preliminary stage of ankle OA, particular varus type^2,11^.

The present results are also similar to previous findings demonstrating that bone resorption in the antero-medial area of the talar trochlea articular surface occurs in ankle OA, which results in a larger radius of the circle approximating the shape of the talar trochlea on the sagittal plane^5,6^. Takakura *et al*.^10^ reported that the loading stress was concentrated on the medial side of the talar trochlea in the severe stage of varus ankle OA, because a varus position of the ankle joint is not adequately compensated by a valgus position of the subtalar joint. The concentration of loading stress induces the damage to cartilage and subchondral bone in that area. Thus, the medial bone resorption of the talar trochlea was remarkable on the deviation color maps in stage 4 ankle OA.

The color map analysis demonstrated that osteophytes are produced on the lateral wall of the talus, particularly in the severe stage of ankle OA (Figs. 3 and 4). Several studies have shown that a distal opening of the medial malleolar articular surface was formed in severe varus ankle OA, resulting in instability of the talus in the ankle mortise^3,10,14^. Since the anteromedial trochlear articular surface usually wears away in varus OA, we hypothesize that ankle OA patients probably support their weight more on the lateral side of the ankle to reduce pain during standing and walking. Therefore, the osteophyte formation on the lateral articular surface of OA tali is likely produced due to accumulation of large loading stress acting on the lateral articular surface. This osteophyte around the joint, if marginal, may also contribute to stabilizing the joint with instability^15,16^. The detailed mechanism underlying the generation of the osteophytes on the lateral wall of the talus should be investigated in future studies.

The present study demonstrated that the degree of morphological change in the talus does not necessarily correspond to the degree of severity of OA according to the Takakura classification; the deviation does not increase linearly as the severity increases, as in Fig. 5. This is possibly due to the fact that the Takakura classification for moderate to severe ankle OA is made based solely on the joint space of the talocrural joint evaluated using weight-bearing radiographs^10^, but not the detailed morphological changes of the talus. The present study showed that the pattern of the morphological degeneration of the talus could be different from patient to patient, even in patients classified as the same stage.

Only a minor morphological difference between the OA and non-OA tali was observed on the talocalcaneal joint surface even in the advanced stage. The subtalar joint is known to compensate for the deformities of the ankle joint due to varus ankle OA^4,17^. However, this compensatory motion of the subtalar joint is probably much smaller than the excessive motion of the pathological talocrural joint^18^ in patients with varus ankle OA, suggesting that the change in the loading stress on the subtalar articular surfaces is probably minor compared to that on the talocrural joint surface in patients with varus ankle OA. This must be confirmed in future studies.

Quantitative understanding of the pattern of talar bony degeneration due to ankle OA is clinically very important, particularly for foot and ankle orthopedic surgeons who perform total ankle arthroplasty for varus ankle OA. In total ankle arthroplasty, a cutting guide is used to determine the cutting level of the talar dome, but the guide is usually placed in the direction parallel to the trochlea articular surface to maintain surface congruity^19,20^, even in the case of varus ankle OA patients^21,22^ whose trochlea articular surface is medially tilted, as observed in Figs. 2–5. This possibly results in misalignment of the talus with respect to the anatomical axis of the tibia. Therefore, several surgeons have recommended resection of the lateral margin of the trochlea prior to placement of the cutting guide, to take the medial surface resorption into account^23,24^. The present results would help determine how the resection of the lateral margin of the trochlea should be made prior to placement of the cutting guide, possibly resulting in better clinical outcomes of total foot arthroplasty. Furthermore, the present study demonstrated that osteophytes are produced on the lateral wall of the talus, particularly in the severe stage of ankle OA. Therefore, it might be beneficial to surgically resect such osteophytes not only on the articular surface of the fibular malleolus, but also on the lateral facet of the talus for correct alignment of the talus with respect to the tibia. Various surgical techniques have been proposed for neutral alignment of the talus in varus ankle OA^21,24–26^, and debridement of the osteophyte on the articular surface of fibular malleolus is often recommended^27^. However, the present study indicated that debridement of the osteophytes on the lateral facet of the talus might also be necessary for correct alignment of the talus with respect to the tibia in total ankle arthroplasty and arthrodesis.

The present study has some limitations. The method used can be applied only to patients with unilateral ankle OA, but not to patients with bilateral ankle OA, since the non-OA side is used as the baseline for quantification of degeneration of the talus. However, this is indispensable to quantitatively clarify the pattern of morphological degeneration of talar morphology while eliminating the effects of the large inter-individual variabilities of talar morphology. Another limitation is the statistical treatment of the results. In the present study, the mean deviations were calculated to quantify the change in the talar morphology, but the mean deviation color map of the whole talar surface across patients could not be calculated because the shape of the talus has large individual variability. For this, the talus of each participant must be transformed (warped) onto a standard template talus using a deformation function. It has been proposed that intra-individual variability of human talar shape is quite small^28^, possibly indicating that the calculation of such a deformation function is quite feasible. On the other hand, some studies have suggested intra-individual variation of the talar shape is considerably large due to sex and age^29,30^. Therefore, tools must be developed to enable detailed statistical analysis of the results obtained using the proposed technique.

## Conclusion

In conclusion, the morphological bony degeneration of the talus due to OA was characterized visually as osteophyte formation and surface resorption around the talar trochlea using a method to compare the left and the right tali in patients with unilateral varus ankle OA. These findings may be due to a stereotypical pathological loading condition of the talus in varus ankle OA and may contribute to better understanding of the development of varus ankle OA. Furthermore, this morphological information will be useful for developing better techniques and designs for total ankle arthroplasty and arthrodesis.

## Methods

### Patient selection

Thirty-five patients (average age, 70.1 years; range, 41 to 88 years; 8 males, 27 females), consisting of an ankle OA group and a control group, were recruited in this study. The ankle OA group included 22 patients (71.1 years; 49 to 88 years; 5 males, 17 females) diagnosed with unilateral varus ankle OA. Varus ankle OA was judged based on a talar medial tilt angle > 4° measured on weight-bearing anterior-posterior radiographs^18^, and stage > 3a according to the Takakura classification. Patients with ankle OA following fractures of the tibia, ankle, or talus or inflammatory diseases such as rheumatoid and infectious arthritis were excluded. Both secondary ankle OA patients following repetitive ligamentous injuries and primary ankle OA patients with unknown sprain histories were included in the analysis. The Takakura classification^10^ of the recruited ankle OA patients was independently evaluated by two of the authors, who are foot and ankle orthopedic surgeons with more than 10 years of experience (H.S. and T.K.), and the classification results were completely identical. The control group included 13 patients (68.5 years; 41 to 84 years; 3 males, 10 females) with fresh trauma without involvement of the talus, such as fractures of the tibia, calcaneus, or forefoot. The diagnostic process with interviews confirmed all patients to be free of any foot and ankle pathologies except for the above fresh trauma. The present study was approved by the Institutional Review Board of Tachikawa Hospital (Tokyo, Japan), and informed consent was obtained from all patients. The methods were carried out in accordance with the approved guidelines.

### Bone model reconstruction

**Figure 6:**
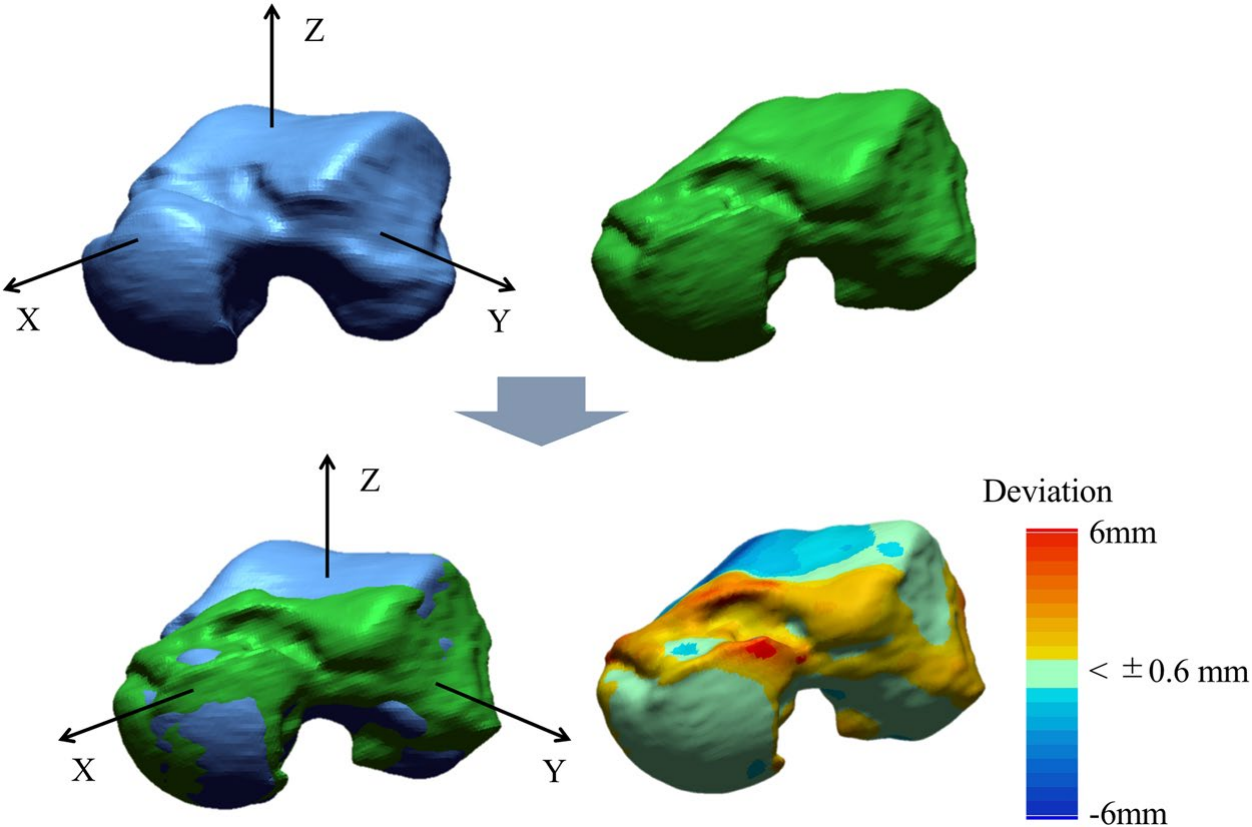
Left-right comparisons of the talus in unilateral varus ankle osteoarthritis. Mirror image models are created for the right-side specimens, and the osteoarthritic talus model (green) is registered to the opposite non-OA talus model (blue) via an iterative closest point algorithm. The surface deviation is calculated as the distance between the two surfaces along the surface normal direction of the non-OA talus. The red and blue colors are deviations of the osteoarthritic talus outside and inside of the non-OA talus.

CT scan data of the hindfeet of the OA and control subjects were obtained in this study using a CT scanner (Aquilion 64, Toshiba Medical Systems Corporation, Otawara, Japan). CT scans of both feet of each participant were performed. All scan data had an in-plane resolution of 512 × 512, with slice thickness of 0.5 mm. CT images were reconstructed at 0.5-mm intervals with pixel size ≤0.682 mm. The left and right tali were then reconstructed in a virtual three-dimensional space using specialized software (Avizo 9.1; FEI Visualization Sciences Group, Hillsboro, OR, USA). Mirror image models were created for the right-side specimens, so that all specimens could be treated as left-side specimens. To facilitate visual comparisons, a body-fixed coordinate system of the talus (the x-, y- and z-axes representing the anteroposterior, mediolateral, and dorsoplantar axes, respectively) was defined based on Lisowski *et al*.^31^ and Kanamoto *et al*.^32^ (Fig. 6).

### Surface deviation analysis

To extract possible left-right differences in the talar morphology of each participant, the OA talus model was registered to the opposite non-OA talus model via an iterative closest point (ICP) algorithm (Fig. 6), using Geomagic^®^ XOS (3D Systems Corporation, Rock Hill, SC, USA). The ICP algorithm iteratively transforms the OA talus model to match the non-OA talus model so as to minimize the distance between the two sets of vertices constituting the two models. The surface deviation was calculated as the distance between the two surfaces along the surface normal direction of the OA talus. The light green color indicated that the deviation was less than 0.6 mm, whereas the red and blue colors were deviations of the OA talus outside and inside of the non-OA talus. The gray color indicated the area where the surface deviation could not be calculated because there was no corresponding surface on the non-OA talus due to large deformation.

To statistically evaluate the differences in talar morphology between the two groups, 14 regions of interest on the articular surfaces of the non-OA talus were defined (Fig. 5), and mean deviations were calculated. Each region of interest was defined as the surface area within a radius of 1 mm from the corresponding landmark digitized on the talus. For each vertex on the OA talus within the sphere, the closest distance to the surface of the OA talus was calculated^33^. The sign of the distance was determined based on the direction of the vertex normal vector (the average of the combined facet normal vectors of all connected polygons using the surface area of each face as the weight). The mean deviations were calculated for each region to quantify and statistically evaluate the degrees of surface growth or resorption observed on the OA talus with respect to the non-OA talus. The color maps of the mean deviations were also provided for each of the three stages (Fig. 5b–d).

### Statistics

A normal distribution was ensured using the Shapiro-Wilk tests. Comparisons between the control and OA groups were performed using the independent *t*-test and the Mann-Whitney U test for normally and non-normally distributed data, respectively, and Pearson’s chi-squared test for categorical variables. The level of significance was p < 0.05. SPSS software (IBM SPSS Statistics Version 23; IBM, Armonk, NY, USA) was used for statistical analyses. A post hoc power analysis was conducted using G*Power 3^34^.

## Data Availability

The authors declare no restrictions on the availability of material and data to the publishing team.
