# Author Correction: Visualization and quantification of the degenerative pattern of the talus in unilateral varus ankle osteoarthritis

**DOI:** 10.1038/s41598-020-60514-4

**Published:** 2020-02-19

**Authors:** Hiroyuki Seki, Naomichi Ogihara, Tetsuro Kokubo, Yasunori Suda, Ken Ishii, Takeo Nagura

**Affiliations:** 1 Department of Clinical Biomechanics, Keio University, 35 Shinano-machi, Shinjuku-ku, Tokyo 160-8582 Japan; 2 Department of Orthopeadic Surgery, International University of Health and Welfare Mita Hospital, 1-4-3 Mita, Minato-ku, Tokyo 108-8329 Japan; 3 Department of Orthopeadic Surgery, School of Medicine, International University of Health and Welfare, 4-3 Kouzunomori, Narita-shi, Chiba 107-0062 Japan; 4 Department of Mechanical Engineering, Faculty of Science and Technology, Keio University, 3-14-1 Hiyoshi, Kohoku-ku, Yokohama-shi, Kanagawa 223-8522 Japan; 5 Department of Biological Sciences, Graduate School of Science, The University of Tokyo, 7-3-1, Hongo, Bunkyo-ku, Tokyo 113-0033 Japan; 6 Department of Orthopeadic Surgery, Tachikawa Hospital, 4-2-22 Nishiki-cho, Tachikawa-shi, Tokyo 190-8531 Japan; 7 Department of Orthopeadic Surgery, International University of Health and Welfare Shioya Hospital, 77 Tomita, Yaita-shi, Tochigi 329-2145 Japan

Correction to: *Scientific Reports* 10.1038/s41598-019-53746-6, published online 25 November 2019

The Acknowledgements section in this Article is incomplete.

“The authors are grateful to Dr. Masateru Kitashiro for his technical assistance with C.T. and two anonymous reviewers for the constructive comments and suggestions.”

should read:

“The authors are grateful to Dr. Masateru Kitashiro for his technical assistance with C.T. and two anonymous reviewers for the constructive comments and suggestions. This work was funded by Ministry of Health, Labour and Welfare (17H01452).”

